# CDK11^p58^ Is Required for Centriole Duplication and Plk4 Recruitment to Mitotic Centrosomes

**DOI:** 10.1371/journal.pone.0014600

**Published:** 2011-01-31

**Authors:** Nathalie Franck, Emilie Montembault, Pierre Romé, Aude Pascal, Jean-Yves Cremet, Régis Giet

**Affiliations:** UMR6061-CNRS, Institut de Génétique et Développement de Rennes, Université de Rennes I, Rennes, France; University of Birmingham, United Kingdom

## Abstract

**Background:**

CDK11^p58^ is a mitotic protein kinase, which has been shown to be required for different mitotic events such as centrosome maturation, chromatid cohesion and cytokinesis.

**Methodology/Principal Findings:**

In addition to these previously described roles, our study shows that CDK11^p58^ inhibition induces a failure in the centriole duplication process in different human cell lines. We propose that this effect is mediated by the defective centrosomal recruitment of proteins at the onset of mitosis. Indeed, Plk4 protein kinase and the centrosomal protein Cep192, which are key components of the centriole duplication machinery, showed reduced levels at centrosomes of mitotic CDK11-depleted cells. CDK11^p58^, which accumulates only in the vicinity of mitotic centrosomes, directly interacts with the centriole-associated protein kinase Plk4 that regulates centriole number in cells. In addition, we show that centriole from CDK11 defective cells are not able to be over duplicated following Plk4 overexpression.

**Conclusion/Significance:**

We thus propose that CDK11 is required for centriole duplication by two non-mutually-exclusive mechanisms. On one hand, the observed duplication defect could be caused indirectly by a failure of the centrosome to fully maturate during mitosis. On the other hand, CDK11^p58^ could also directly regulate key centriole components such as Plk4 during mitosis to trigger essential mitotic centriole modifications, required for centriole duplication during subsequent interphase.

## Introduction

The centrosome of somatic cells is the main microtubule organising center [1]. It is required to organise the cytoplasmic microtubule network during interphase and the mitotic spindle during mitosis. This organelle consists of two centrioles embedded in an amorphous pericentriolar material (PCM). In proliferating cells before division, the centrosome needs to be duplicated precisely once so that the mitotic cell harbours two centrosomes, each comprising two centrioles. These two centrosomes will be used to nucleate the microtubules required to assemble the mitotic bipolar spindle during mitosis [2]. The centrosome duplication cycle is divided into several key steps. First, during mitosis, the two paired-centrioles inherited by the daughter cell lose their orthogonal configuration, a process called disengagement. Then, procentrioles (daughter centrioles) nucleate in G1/S phase at an orthogonal angle next to each mother centriole. The two newly synthesised centrioles elongate during the S and G2 phases. In late G2, as cells prepare for mitosis, centrosomes increase in size and recruit additional PCM to enhance their ability to nucleate microtubules. This process is referred to as centrosome maturation. At the end of the G2 phase, the two newly duplicated centrosomes separate to organise a bipolar mitotic spindle, enabling each daughter cell to inherit one centrosome after cell division.

In contrast to normal cells, tumour cells frequently show centrosome number defects that are thought to be the consequence of abnormal regulation of the centriole duplication machinery. Thus, the restriction to a single round of centriole duplication per cell division cycle contributes to the prevention of aberrant centrosome numbers, multipolar spindles and chromosomal instability [3], [4].

Several proteins are critical for the canonical centriole assembly. Based on genome-wide RNAi and genetic screens in *C. elegans*, a molecular hierarchy for the recruitment of five essential proteins for centriole assembly has been established. SPD-2 is necessary to recruit ZYG-1 at the centriole. Then, both proteins in turn recruit SAS-5 and SAS-6, followed by SAS-4 [5], [6], [7]. Most of these proteins have conserved roles in centriole duplication in other systems. Cep192, the human homolog of *C. elegans* SPD-2 protein, is a pericentriolar protein required for both centriole duplication and centrosome maturation [8], [9]. Plk4, related to *C. elegans* ZYG-1, as well as CPAP and HsSAS-6, human homologs of C. *elegans* SAS-4 and SAS-6, have been identified as key regulators of centriole duplication [8], [10], [11], [12]. For example, overexpression of Plk4 leads to centriole amplification; conversely, mutations or inhibition of the *DmSAK/PLK4* gene dramatically impairs centriole duplication [10], [11]. Of these proteins, Plk4 is the only enzyme. It is a short-lived protein kinase that localises at centrioles throughout the cell cycle. Its degradation and activity are tightly regulated during the cell cycle, these two processes being crucial for the centriole biogenesis process [13], [14].

SAS-6, Chk2, CDC25c and Hand1 remain the only identified substrate of Plk4/SAK/ZYG-1 [15], [16], [17], [18], [19], [20]. The physiological relevance of CDC25c and Chk2 phosphorylation events by Plk4 is unknown. SAS-6 phosphorylation by ZYG-1 in *C. elegans* triggers its targeting to the newborn centriole. The Plk4-mediated phosphorylation of the Hand1 transcription factor inhibits its sequestration in the nucleolus. Hand1 is then released in the nucleus where it activates the transcription of genes required for cell differentiation. Interestingly, a growing list of studies in different models shows that Plk4 centrosomal levels and activity are maximal during mitosis, suggesting the kinase also plays a key role during mitosis, possibly to licence the centriole for subsequent duplication in S phase. However, this possible role remains speculative and difficult to challenge experimentally [14], [21].

Cyclin-dependant kinase 11 (CDK11) mRNA produces a 110 kDa isoform protein expressed throughout the cell cycle and a 58 kDa isoform protein which is specifically translated from an internal ribosomal entry site sequence during G2/M [22]. The small isoform has been shown to be an essential regulator of mitosis. Indeed, CDK11^p58^ is required for centrosome maturation, bipolar spindle assembly, maintenance of sister chromatid cohesion and cytokinesis [23], [24], [25].

In this article we describe a new role for CDK11^p58^ in the duplication of centrioles. Our data reveal that CDK11 is necessary to ensure proper centriole duplication. Furthermore, we identify Plk4 as a novel partner of CDK11^p58^ and show that CDK11^p58^ depletion impairs the proper recruitment of Plk4 to mitotic centrosomes.

## Results

**Figure 1:**
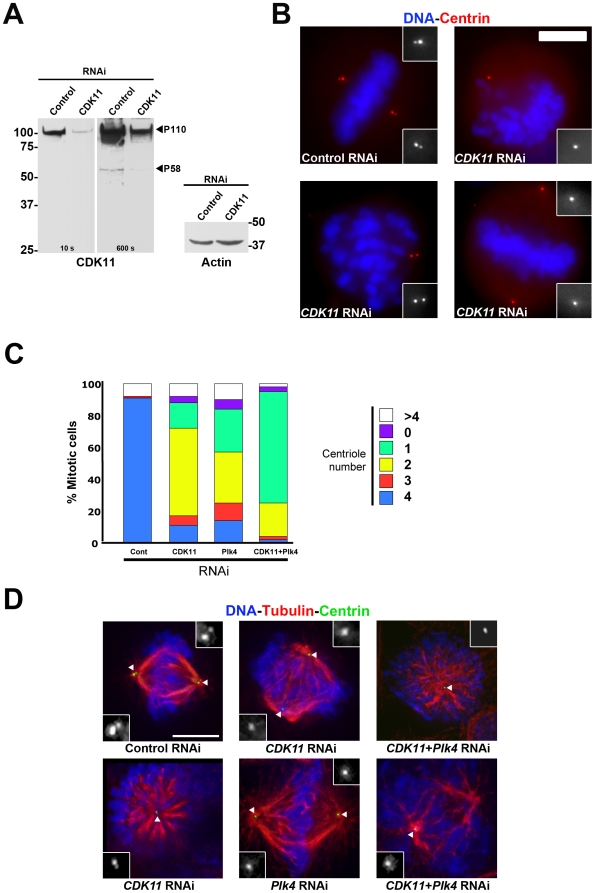
CDK11^p58^ depletion leads to a reduction in centriole number in mitotic HeLa cells. Mitotic HeLa cells stably expressing a GFP-tagged centrin were subjected to control or *CDK11* RNAi and analysed after 72 hours. A) Western blots showing CDK11^p110^ and CDK11^p58^ (with 10 and 600 seconds exposure times respectively) and actin protein levels are shown. The positions of CDK11^p110^ and CDK11^p58^ are indicated. B) Mitotic cells stained for DNA (blue) and centrin (red) following control or *CDK11* RNAi. Some of the observed defects are shown here. The insets show a 3× magnification view of the centriole region in monochrome. The treatment is displayed at the bottom of each panel. Scale bar is 5µm. C) Quantitative analysis of the centriole distribution following control, *CDK11*, *Plk4* or double RNAi in mitotic cells. See the reduced number of centrioles following *CDK11* RNAi (also shown in Table S1). D) Example of the mitotic figures observed in control, *CDK11*, *Plk4* and double (*CDK11* and *Plk4*) siRNA–transfected cells. See also detailed analysed in Table S1. Centrioles are green, microtubules are red and chromosomes are blue. The insets show a 3× magnification view of the centriole region in monochrome (indicated by a white triangle in the merge panels). Bar is 5µm.

We have shown previously that CDK11^p58^ is required for centrosome maturation (Petretti et al., 2006). A growing list of studies reports that failure to properly orchestrate centrosome maturation can trigger centriole duplication defects. As an example, Cep192 is a major regulator of both centrosome maturation and centriole duplication in *C. elegans* and in mammalian cells [7], [8], [26]. These data prompted us to investigate whether CDK11^p58^ was also required for centriole duplication. To do so, we first looked at the effects of a three-day siRNA-mediated depletion of CDK11 on centriole numbers in asynchronously growing mitotic cells (Figure 1 A). HeLa cells stably expressing GFP-centrin protein [27] were transfected with control or *CDK11* specific siRNAs [24] and centrioles were quantified on mitotic figures after fixation of the cells (Figure 1 D). As expected, 90.6% of control metaphase cells showed four centrioles with two centrin dots at each pole of the mitotic spindle (Figure 1 and Table S1). By contrast, after depletion of CDK11, as shown previously, only 37.9% of the spindles were bipolar [24]). Amongst these, only 10.8% of the cells looked normal with two centrin dots at each pole whereas 26.9% of the cells showed spindles with less than two centrioles at one pole. The remaining mitotic cells appeared to form very short or monopolar spindles (54.4%) and all of them showed a reduction in centriole numbers (i.e. less than four centrioles in total). *Plk4* RNAi treatment, used as a positive control (Figure 1 C, D and Table S1), yielded highly similar phenotypes with only 14.4% of the cells showing bipolar mitotic spindles with four centrioles, two at each pole. The vast majority of the mitotic cells displayed monopolar and disorganised spindles and showed less than four centrioles. Interestingly, co-depletion of Plk4 and CDK11^p58^ proteins resulted in an 8- to 9-fold decrease of the number of cells showing bipolar spindles with four centrioles (10.8% and 14.4% respectively in CDK11- and Plk4-depleted cells *vs.* 1.5% in co-depleted cells). We observed a dramatic increase in the number of cells with only one centriole when both CDK11 and Plk4 were knocked down (13,9% and 17.8% respectively in CDK11 and Plk4-depleted cells vs. 67.8% in co-depleted cells) (Figure 1 C, D and Table S1).

**Figure 2:**
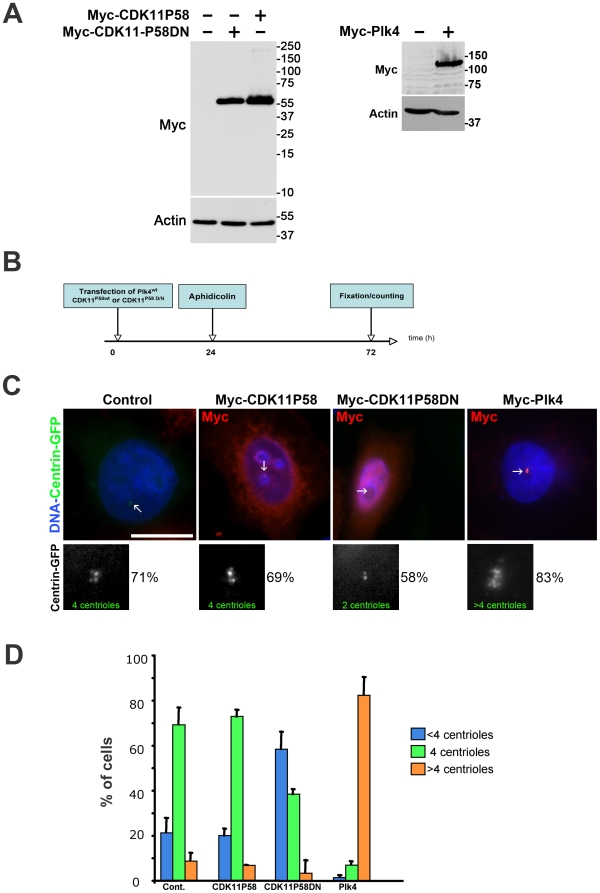
Kinase-dead CDK11^p58D/N^ but not wild-type CDK11^p58^ overexpression leads to a centriole duplication failure in S-phase arrested HeLa cells. A) Expression of Myc-CDK11^p58^ and Myc-CDK11^p58D/N^ (left) or Myc-Plk4 proteins following transfection of the appropriate constructs. A Myc and an actin (as a loading control) Western blotting are shown. B) HeLa cells stably expressing GFP-tagged centrin were transfected with either active (CDK11^p58^) or inactive (CDK11^p58DN^) CDK11^p58^ expression constructs. A positive control was also performed using Myc-Plk4. After 24 hours (duration of one cell cycle), the cells were arrested in S phase by aphidicolin treatment for 24 hours and the centrioles were counted after 48 more hours. C) The transfected cells expressing Myc-tagged proteins were visualised using an anti-Myc antibody (red). DNA is blue. The centriole (green), indicated by arrows, (also displayed in monochrome in the lower panels) were counted in control or transfected cells. In cells expressing CDK11^p58^ as well as in control cells, the vast majority of the cells (∼70%) displayed four centrioles. This percentage dropped to 38.2% when CDK11^p58DN^ was expressed. Note that more than 80% of the Plk4 over expressing cells showed centriole amplification. Scale bar is 10µm. D) Graph (±SD) showing the percentage of cells with <4 centrioles (blue), 4 (green) or >4 (orange).

Most of the proteins required for centrosome duplication localise to this structure. To localise CDK11^p58^ protein kinase within the cell, we decided to generate a HeLa “Tet-ON” cell line which allows the temporally controlled expression of Myc-tagged CDK11^p58^ (Figure S1). The Myc antibody readily recognised one protein of the expected size in the above cell line induced to express the transgenic protein (data not shown) and also in transiently transfected cells (Figure 2 A) but did not reveal any specific bands in non-induced or control cells validating our Tet-inducible expression system. Immunofluorescence experiments were performed after a very short period of induction (1 to 2 hours), when the protein was expressed at low level. We found that Myc-tagged CDK11^p58^ protein kinase was cytoplasmic in interphase (Figure S1 A and B). During early prophase, CDK11^p58^ was principally detected around the separating centrosome and on newly nucleated microtubules. In metaphase cells, the protein localised in the vicinity of the centrosome, and decorated spindle microtubules (Figure S1 and C). Following depolymerisation of microtubules by cold treatment, no obvious localisation of CDK11^p58^ was observed at the mitotic centrosomes suggesting that the centrosomal localisation is microtubule dependent (data not shown). CDK11^p58^ persisted on microtubules emanating from the poles and on the central spindle during telophase and was finally enriched on both sides of the midbody during cytokinesis (Figure S1). Interestingly enough, longer induction to express high levels of Myc-tagged protein increased the cytoplasmic pool resulting in the masking of the signal on mitotic microtubules. This latter result might suggest the existence of a limited number of CDK11^p58^-binding sites on the mitotic spindle.

The RNA interference of known regulators of centriole duplication leads to a decrease of the centriole number. By contrast, only a very low number of these molecules, including the Plk4 protein kinase, triggers centriole amplification *via* their overexpression (reviewed in Bettancourt 2009). To continue the investigation on the function of CDK11^p58^ in centriole biogenesis, we decided to check whether the overexpression of a Myc-tagged wild-type CDK11^p58^ protein, a kinase-dead variant (CDK11^p58DN^) and Plk4 (as a positive control) could modify centriole duplication (see Figure 2 A). CDK11^p58^, CDK11^p58DN^, Plk4 were transiently expressed in HeLa cells stably expressing the GFP-centrin protein. A day after transfection, aphidicolin was added for 48 hours on these cells to arrest them in S phase (Figure 2 B). Cells were then immuno-stained with Myc antibody and the centrioles of Myc-positive arrested cells were counted (Figure 2 C). As expected, the vast majority (71%) of control cells had duplicated their centrioles during this arrest and thus exhibited four centrioles per cell (two pairs of two closely-linked centrioles). When compared to controls, cells overexpressing CDK11^p58^ did not show significant differences in their centriole numbers (∼70%, Figure 2 D). By contrast, the overexpression of the catalytically inactive CDK11^p58DN^ caused a decrease in the number of cells with four centrioles. Indeed, only 38% of these cells contained four centrioles, while 58% failed to duplicate their centrioles, resulting in the presence of cells with either two or three centrioles (Figure 2 C and D). In a parallel assay, the ability of these cells to amplify their centrioles after Plk4 overexpression was assessed. As published before [11], most of the Myc-Plk4 positive cells amplified their centrioles (Figure 2 C, right panels, and D). Therefore, the dead variant of CDK11^p58^ has the ability to inhibit centriole duplication, suggesting it behaves as a dominant negative toward the endogeneous active kinase to prevent centriole duplication. CDK11^p58^ kinase activity would thus be required for this process.

In order to further corroborate our observation that CDK11^p58^ is required for centriole duplication, we used a centriole overduplication assay in U2OS cells. In this cell line, a prolonged S phase arrest induces multiple rounds of centriole duplication. As a consequence, inhibition of proteins required for centriole duplication leads to a decrease in the number of cells with more than two centrosomes (Habedanck, 2005). U2OS cells were transfected with control, *Plk4* (as a positive control) or *CDK11* siRNA for 24 hours before being arrested during S phase with aphidicolin for 48 hours (Figure S2 A). Cells were fixed and centrosomes were stained with anti-γ-tubulin antibodies (Figure S2 B). In control S-phase arrested cells, 29.0±1.4% of the cells showed more than two centrosomes (Figure S2 C). A noticeable decrease was observed in Plk4 and CDK11-depleted cells, with a percentage of cells displaying more than two centrosomes of 9.0±2.1% and 11.9±1.7% respectively, indicating a ∼3-fold inhibition of the spontaneous centriole over duplication process observed in U2OS control cells. This latter result further highlights the requirement of CDK11^p58^ in the centriole duplication process.

**Figure 3:**
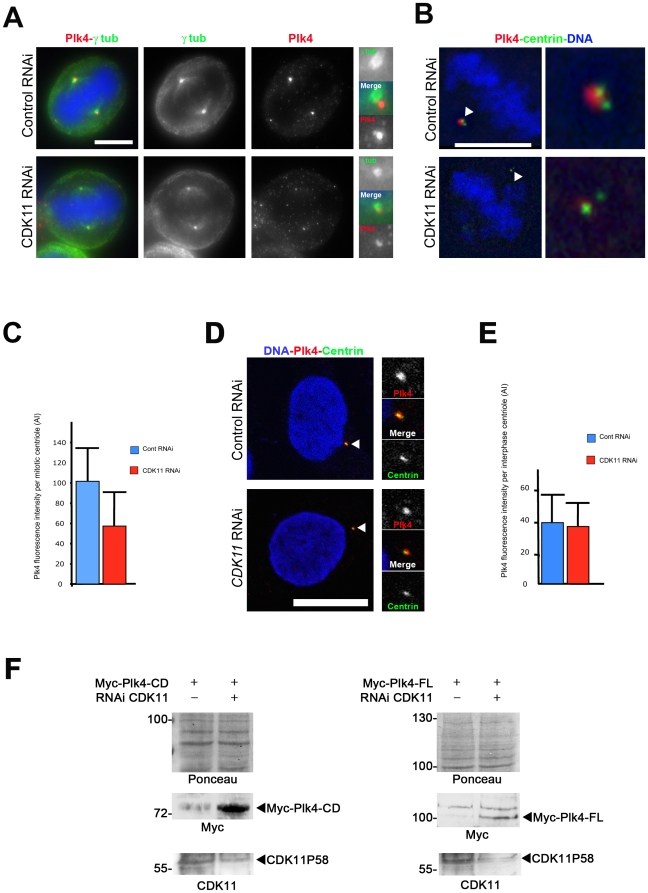
CDK11 depleted cells show diminished recruitment of the Plk4 protein at the mitotic centrosomes. A) HeLa cells were transfected with control or *CDK11* siRNAs and stained for Plk4 (red and right panels in monochrome) and γ tubulin (green and middle panels in monochrome). In the absence of CDK11^p58^, Plk4 signal is strongly reduced at the centrosome of mitotic cells. For each cell, one of the two centrosomal regions is enlarged on the left panel. Bar is 5µm. B) Single section of a control or *CDK11* siRNA treated cell stained for centrin (green) and Plk4 (red). The insets show a 5× magnification of the spindle pole region that contains 2 centrioles. See the strong reduction of the Plk4 signal in the CDK11–depleted cell compared to the wild-type cell. Bar is 5µm. C) Graph showing the significant difference (p<0,0005) of Plk4 signal intensity (±SD) per mitotic centriole in control (blue) or *CDK11* siRNA (red) transfected cells. D) Control (top) or *CDK11* (bottom) siRNAs-transfected HeLa interphase cells stained for Plk4 (red), centrin (green). The right panels show a 3× magnification of the centrosomal region. Bar is 10µm. E) Graph showing the Plk4 signal (±SD) intensity per interphase centriole in control (blue) or *CDK11* siRNA transfected cells (red). F) Analysis of exogenous Plk4 protein levels in CDK11 knock down cells. Myc-Plk4-CD (Catalytic Domain; amin-acids 1–638, left panels) and Myc-Plk4-FL (Full Length; amino-acids 1–970, right panels) expressing constructs were co-transfected with control or *CDK11* siRNAs and the CDK11^p58^ (bottom panels) and Myc-Plk4 (middle panels) proteins levels were analysed by Western blotting 48 h following transfection. The membrane was stained by Ponceau S (top panels). In four different experiments, exogenous Plk4 proteins are hardly detectable after 48h hours in control cells whereas they are more stable following *CDK11* RNAi.

Previous experiments showed that CDK11-depletion diminished γ-tubulin and Plk1 recruitment at the centrosomes during centrosome maturation [24]. Interestingly, Plk1 depletion abolishes Cep192 recruitment at centrosomes [8], [26]. We first decided to analyse the levels of Cep192 protein at mitotic centrosomes (containing 2 centrioles) following control or *CDK11* RNAi (Figure S3 A). Quantification of the signal revealed that centrosomal Cep192 was reduced by 3-fold at centrioles in mitotic CDK11-depleted cells (Figure S3 B) but the interphase centrosome level was unchanged (data not shown). SPD-2, the worm counterpart of Cep192 is also required for ZYG-1, the related orthologue of Plk4, to be recruited at centrosomes [5], [7]. Plk4 is a centriole-associated protein kinase. The protein kinase levels in the whole cell and at the centrosome are elevated during mitosis, by contrast to interphase. We next monitored whether CDK11^p58^ was also implicated in Plk4 recruitment to the centrosomes in mitosis. Control and *CDK11*-RNAi treated cells were stained with Plk4, centrosome (Figure 3 A) or centriole probes antibodies (Figure 3 B). Strikingly, CDK11 depletion also impaired Plk4 accumulation at mitotic centrosomes (Figure 3 A–C) whereas staining of Plk4 at centrosomes in interphasic cells was not reduced (Figure 3 D and E). In parallel, we also checked whether the Plk4 protein was stable in CDK11 knock down cells to rule out the possibility that the low amount of centrosomal Plk4 after *CDK11* RNAi was caused by an overall Plk4 protein disappearance. As endogenous Plk4 protein cannot be detected by Western blot, we examined the stability of full length Plk4 or a shorter Plk4 fragment also containing its phosphodegron motif [13]. For this purpose, we co-transfected control or *CDK11* siRNA together with the Myc-tagged Plk4 constructs and examined by Western blot exogenous Plk4 protein levels. Exogenous Plk4 proteins were very unstable and difficult to detect in the control cells 48 h following RNAi. However, we found that Myc-Plk4 proteins were more stable in CDK11 knock down cells (Figure 3 F), suggesting that more Plk4 protein was present in this case but not targeted to the mitotic centrosomes. Taken together, the above results point out that CDK11^p58^ is essential for the correct recruitment of multiple centrosomal proteins including Plk1 and γ-tubulin, but also Cep192 and Plk4.

**Figure 4:**
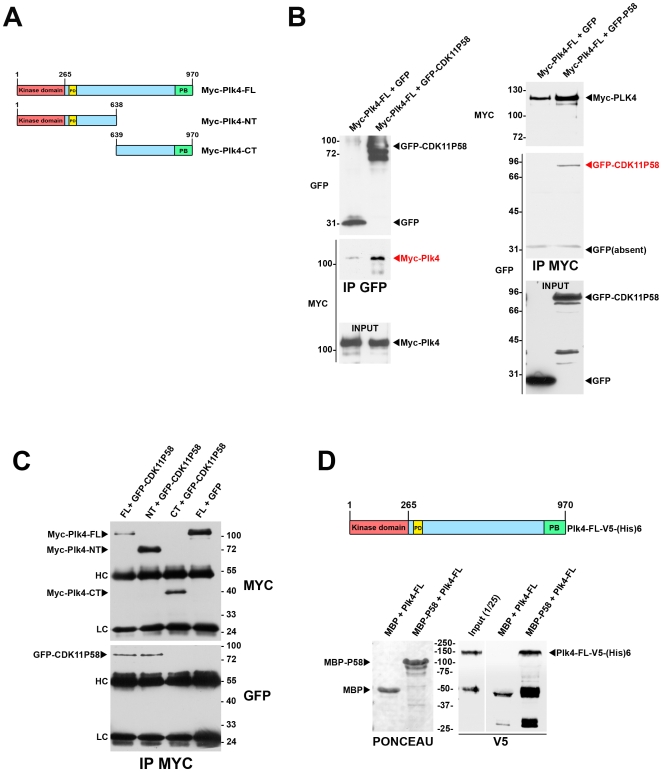
Plk4 and CDK11^p58^ can interact *in vitro* and *in vivo*. A) Scheme of the different Plk4 constructs used for the co-transfection and immunoprecipitation experiments. Myc-Plk4-FL: Myc-tagged Plk4 full length; Myc-Plk4-NT: Myc-tagged Plk4 N-terminus domain; Myc-Plk4-CT: Myc-tagged Plk4 C-terminus domain. The catalytic domain (CD) is displayed in red, the polo box (PB) in green and the phosphodegron (PD) in yellow. B) GFP or GFP-CDK11^p58^ were co-transfected with Myc-Plk4 in COS7 cells and the proteins were immunoprecipitated using GFP (left) or Myc antibodies (right). Myc-Plk4 was co-immunoprecipitated with GFP-CDK11^p58^ but not with GFP (left). GFP-CDK11^p58^ (but not GFP) was also found in the Myc-Plk4 immunoprecipitates (right). C) Myc-Plk4-FL, Myc-Plk4-NT, Myc-Plk4-CT were co-transfected with GFP and/or GFP-CDK11^p58^ in COS7 cells. Myc-tagged Plk4 proteins were immunoprecipitated and revealed by Myc (top)(bottom). GFP-CDK11^p58^ is detected in Myc-Plk4-FL and NT immunoprecipitates whereas it is absent from Myc-Plk4-CT pull down (bottom). HC: immunoglobulin heavy chains; LC: immunoglobulin low chains. D) Maltose Binding proteins (MBP) or MBP-CDK11^p58^ proteins were immobilised on amylose beads and incubated with recombinant Plk4-V5-(His)6. After washes, the beads (and bound proteins) were boiled in Laemmli buffer and the proteins were analysed following EGPA-SDS by Ponceau S staining to visualise MBP and MBP-CDK11^p58^ (left). The membrane was then probed by anti-V5 antibody to reveal the bound Plk4 recombinant protein. The input (1/25th) is also shown on the gel. Thus, Plk4 and CDK11^p58^ can bind directly to each other.

To date, very few proteins have been found to associate with Plk4. We further tempted to investigate whether CDK11^p58^ could physically interact with this master regulator of centriole duplication. As endogenous CDK11^p58^ and Plk4 proteins are difficult to detect in cell lysates, we decided to co-express these two proteins with different tags in COS-7 cells and we carried out immunoprecipitation experiments (Figure 4 A, B and C). We were able to detect Myc-Plk4 in GFP-CDK11^p58^ pull down (IP GFP, Figure 4 B) and GFP-CDK11^p58^ was reciprocally found in Myc-Plk4 pull-down (IP MYC, Figure 4 B). We then performed the same experiment using truncated Myc-Plk4 proteins containing either the N-terminal or the C-terminal end of the protein (Figure 4 C). Interestingly enough, the interaction was only observed between CDK11^p58^ and the N-terminal domain of Plk4 (NT), which contains the kinase catalytic domain and the phosphodegron motif required for protein stability [13]. We didn't find any interaction of CDK11^p58^ with the Plk4 C-terminal fragment containing the polo box (Figure 4 C). To verify that Plk4 and CDK11^p58^ were able to interact directly, we performed an *in vitro* pull down assay using Maltose-Binding Protein (MBP)-tagged CDK11^p58^ immobilised on an amylose column and a recombinant V5-tagged full length Plk4 (See Method section and Figure 4 D). Although the immobilised CDK11^p58^ protein kinase was inactive (not able to phosphorylate any of the *in vitro* CDK kinase substrates like histone H1, MBP or casein), it was able to interact with Plk4 *in vitro*, revealing a direct interaction between the two proteins (Figure 4 D).

As CDK11^p58^ and Plk4 are protein kinases that physically interact, it was tempting to speculate that one could be the substrate of the other. Unfortunately, soluble recombinant CDK11^p58^ is inactive so the CDK11^p58^ kinase assay on Plk4 was not possible. However, we were able to check that active Plk4 could not phosphorylate a recombinant MPB-tagged CDK11^p58^ (Figure S4), since after incubation of these proteins together we couldn't observe any phosphorylated band, whereas Plk4 was able to phosphorylate b casein (positive control). This result suggests that CDK11^p58^ is not a substrate of Plk4.

**Figure 5:**
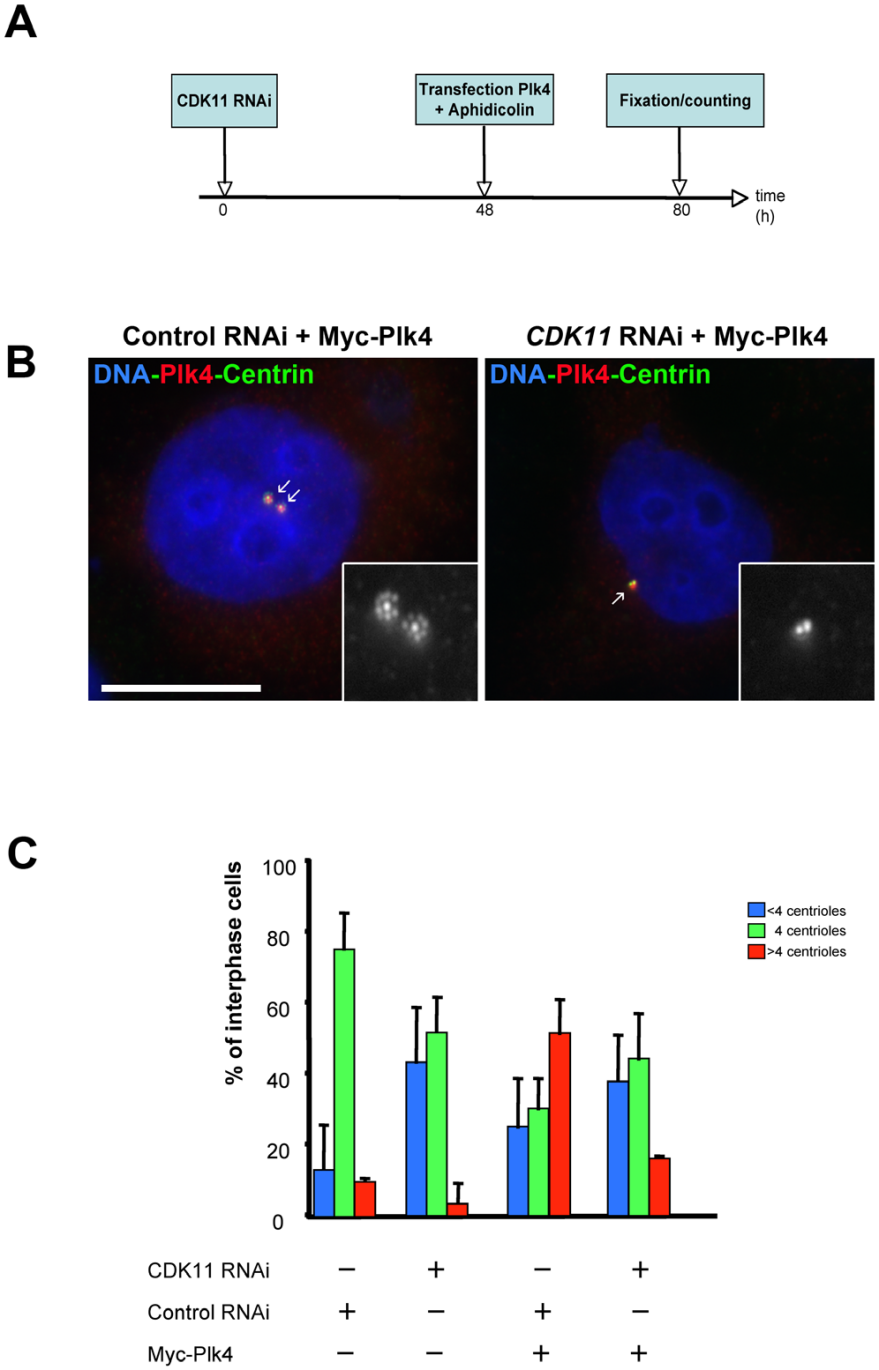
CDK11-depleted cells cannot amplify their centrioles following Plk4 overexpression. A) Control or CDK11-depleted cells were transfected with Myc-Plk4 construct and their ability to amplify their centrioles was analysed after S phase arrest. B) Example of a control siRNA treated cell (left) over expressing Plk4 (red). See the presence of additional centrin (green) dots around each mother centriole. By contrast, most of Myc-Plk4 over expressing cells can not amplify their centrioles following CDK11 siRNA transfection although the over expressed Plk4 protein kinase is strongly recruited to the centriolar region.The insets shows a 5× magnification of the centrosomal region (centrin staining in displayed in monochrome). Bar is 10µm.

Our study reveals that the mitotic specific CDK11^p58^ is able to associate with Plk4 during mitosis and is required to target the Plk4 protein kinase to the centrosome in mitosis. In a last experiment, we decided to check whether mitotic centrioles that had passed mitosis with decreased CDK11 activity were able to respond to high Plk4 activity in the following interphase by amplifying their centrioles. For this purpose, we performed *CDK11* RNAi on HeLa cells for two days (to allow CDK11 depletion) and we overexpressed Myc-Plk4 by transient transfection to trigger centriole amplification in the presence of aphidicolin (Figure 5 A). As expected in S-phase arrested control cells, a majority of the cells (75.9±10.4%) displayed 4 centrioles whereas only 52.1±10.0% of the CDK11–depleted cells exhibited this phenotype (Figure 5 C). In this population, we also observed a strong increase of cells with less than 4 centrioles (43.8±15.6%) compared to controls (13.8±4.9%). When we overexpressed Myc-Plk4 in control siRNA treated cells, we observed a strong increase in the number of cells showing a severe centriole amplification (52.1±9.8%) whereas a limited percentage of CDK11 knock down cells overexpressing Plk4 were able to amplify their centrioles (16.7±0.4%). However, in these interphase-arrested cells, a strong recruitment of Plk4 could be observed (Figure 5 B, right panel). Thus, overexpressed Plk4 protein kinase is strongly recruited to interphase centrioles but cannot induce their amplification following *CDK11* RNAi.

## Discussion

Our study reveals for the first time a role for CDK11^p58^ during the centriole duplication process, and our data suggest that the protein acts in helping the recruitment of master regulators of this process at centrosomes during mitosis. The kinase activity of CDK11^p58^ seems necessary for its function in the centriole duplication process, since the overexpression of the kinase-dead variant phenocopies *CDK11* RNAi and inhibits centriole duplication.

In agreement with such a mitotic role, the CDK11^p58^ isoform is of low abundance and generated through the use of an internal ribosome entry site on the *CDK11* mRNA regulated in a mitosis specific manner, leading to this isoform being only present in mitosis [22].

To explain this new function of CDK11^p58^, two hypotheses can be made (see model Figure S5). First, we have shown previously that CDK11^p58^ is necessary to recruit Plk1 and γ-tubulin during centrosome maturation, which in turn can affect the recruitment of Cep192 and thus possibly Plk4 as well [24]. Indeed, Haren *et al.* have described that Cep192 recruitment was abolished in Plk1-depleted cells [26]. Moreover, the Cep192 worm counterpart, SPD-2, is required for ZYG-1, the related orthologue of Plk4, to be recruited at centrosomes [5], [7]. Furthermore, Plk1 and separase have been recently involved in the licensing mechanism [28], [29]. The “licensing model” suggests that centrioles (like DNA) are marked during mitosis to allow their duplication in the following interphase [2], [14], [30]. In agreement with this “licensing” hypothesis, electron microscopy studies of amplifying centrioles revealed that only the mother centrioles (generated from previous cell cycle) can be used as a template to generate daughter centrioles [6]. After down regulation of Plk1, centrioles fail to disengage and subsequently centriole duplication is blocked [28], [29]. These last results demonstrate that a complex network of proteins might be necessary to trigger the centrosome biogenesis and that a strong correlation exists between maturation of centrosomes during mitosis and their duplication in interphase (Figure S5).

The second hypothesis would be that CDK11^p58^ might also mediate its role *via* its direct interaction with Plk4. However, CDK11^p58^ associates with mitotic microtubules but is not localised at the centrioles itself and was not, so far, characterised as a core component of purified human or *Drosophila* centrosomes [31], [32]. This might suggest that the interaction with Plk4 occurs mostly in the cytoplasm of the cell or on spindle microtubules. Interestingly, the level of Plk4 protein kinase is tightly regulated throughout the cell cycle by ubiquitin-mediated proteolysis via SCF^Slimb^ in *Drosophila* [13], [14] and human cell lines [33]. Plk4 degradation and maintenance of appropriate protein levels in the cell requires phosphorylation of a phosphodegron motif present in the N-terminal fragment of the protein (Figure 4 A). Plk4 itself but possibly other kinases are required for phosphorylation of this important motif [33]. This phosphorylation of the phosphodegron is crucial to allow the binding of SCF^Slimb^ and in turn for the degradation of the protein because defective Plk4 degradation triggers Plk4 stabilisation and centriole amplification [13], [14], [33], [34]. We first hypothesised that Plk4 was a substrate of CDK11^p58^ on this phosphodegron motif. Indeed, we found elevated levels of exogenous tagged-Plk4 proteins following CDK11^p58^ depletion reflecting a possible stabilisation of endogenous Plk4 following *CDK11* RNAi. However, the global effect following *CDK11* RNAi was a centriole duplication defect and not an over duplication as expected in case of Plk4 overexpression. In addition, centrioles from CDK11-depleted cells were not able to over duplicate even if forced Plk4 overexpression was induced. This result suggests that, in CDK11-depleted cells, the centrioles lacked essential mitotic modifications allowing their subsequent duplication in S phase (Figure S5). To conclude, if Plk4 does play a role during mitosis, this possible Plk4 mitotic function cannot be compensated by elevation of Plk4 protein levels during interphase after CDK11 depletion.

To date, CDK11^p58^ was suggested to play key roles at various stages of the cell division, during centrosome maturation, chromatid cohesion, and cytokinesis. Our data show for the first time a new role for CDK11^p58^ during centriole duplication, probably mediated to some extent by the centrosomal direct or indirect recruitment of Plk1 and Plk4 protein kinases at centrosomes during mitosis. The emerging dual function of the Cep192, Plk1 and CDK11^p58^ proteins during centrosome maturation and centriole duplication suggests that these two mechanisms and the machinery involved in these processes are closely connected [8], [26], [28]. Further studies will be required to understand the mechanism of action of CDK11^p58^ to potentially validate key centrosomal proteins as substrates of this protein kinase.

## Materials and Methods

### Plasmid and constructs

CDK11^p58^ and CDK11^p58N^ were amplified by PCR using the oligonucleotides ^5′^TAGAATTCCCAGTGAAGATGAAGAACG^3′^ and ^5′^TAGGATCCCCAGTGAAGATGAAGAACG^3′^ and subcloned into the *EcoRI* and *BamH1* sites of the COM263 pcDNA3.1/3xmycA/TO vector (kind gift of Pr Erich Nigg, University of Basel). Myc-tagged expression constructs were described before and were kindly given to us by Erich Nigg [11]. Plk4 protein was cloned into the pET102 plasmid following the manufacturer instructions (Invitrogen) by PCR using the oligonucleotides ^5′^CACCATGGCGACCTGCATCGGGGAG^3′^ and ^5′^ATGAAAATTAGGAGTCGGATTAGA^3′^ to generate the pET102-Plk4-V5-(His)6 expression vector. pCMV-GFP-CDK11^p58^ was described before [24].

### Cell culture, transfections, siRNA-mediated protein depletion and chemicals

The HeLa, HeLa TRex, and COS-7 cell lines were grown at 37°C in DMEM containing 10% FCS and 100U/ml of streptomycin and penicillin (Invitrogen). The U2OS TRex cell line was grown in DMEM containing 10% tetracycline negative FCS (PAA, A15-109), supplemented with 50µg/ml Hygromycine B (Invitrogen, 10687-010). Tetracycline inducible cell lines expressing Myc-tagged wild type CDK11^p58^ was generated by transfection of U2OS-TRex cells kindly given to us by Pr Erich Nigg [11]. Stable transformants were established by selection for two weeks with 1mg/ml G418 (Invitrogen) and 50mg/ml Hygromycin B. The HeLa cell line carrying a GFP-tagged centrin was cultured in DMEM containing 10% FCS, 100UI/ml of streptomycin and penicillin and 500µg/ml G418 [27]. DNA transfections were performed using Lipofectamine 2000 following the manufacturer instructions (Invitrogen). CDK11 and Plk4 proteins were depleted as already described [11], [35]. For control RNAi, a luciferase siRNA was used (Eurogentec). Cells were arrested in S-phase using aphidicolin (4µg/ml) from *Nigrospora sphaerica* (Sigma, A0781).

### Antibodies

The following antibodies were used in this study: mouse anti-GFP (0,4mg/ml, Roche), mouse anti-myc (clone 9E10, dilution 1µg/ml, Invitrogen), rabbit anti-centrin antibody (dilution 1/1000, Abcam), rabbit anti-pericentrin (dilution 1/10 000, Abcam), mouse anti-γ-tubulin (dilution 1/1000, clone GTU-488, Sigma), rat anti-tubulin (clone YL^1/2^, dilution 1/1000, Millipore), rabbit anti-Cep192 (dilution 1/500, kind gift of Laurence Pelletier, Toronto, Canada), affinity-purified rabbit anti-Plk4 and affinity–purified rabbit anti-CDK11 antibodies [11], [21], [24]. All secondary antibodies used in immunofluorescence microscopy were from Molecular Probes, and the peroxidase-coupled secondary antibodies used for Western blotting were from Jackson Immunoresearch Laboratories.

### Centrosome and centriole countings in fixed cells

Centriole countings in mitotic HeLa cells were performed using a cell line carrying a GFP-centrin transgene. Centrioles were obviously visible as 2 dots at each pole of mitotic control cells. In U2OS interphase cells, centrosomes and centrioles were counted in the cells after g tubulin and/or centrin staining respectively, following a method described previously [6]. Each experiment was repeated 3 times and >100 cells were scored by experiment.

### Immunoprecipitations

5.10^6^ cells expressing the tagged constructs were lysed into Lysis Buffer (LB: 50mM HEPES, 100mM KCl, 1mM MgCl_2_, 1mM EGTA, 0,5% NP40, 1mM β mercapto-ethanol, 20mM β glycerophosphate, anti-protease inhibitor cocktail (Roche)). The cell extracts were centrifuged for 10min at 10 000g and the soluble proteins were incubated with 5µg of anti-GFP or anti-Myc antibodies adsorbed on 50µl of magnetic Dynabeads protein A beads (Invitrogen) for 3 hours. The immunoprecipitated proteins were washed 3 times in LB during 5min. The proteins were subjected to SDS polyacrylamide gel electrophoresis (SDS-PAGE), followed by Western blotting analysis.

### Kinase assays

For Plk4 kinase assays, 100ng of GST-Plk4 (Ozyme) was incubated with b casein or Maltose Binding Protein (MBP) or MBP-tagged CDK11^p58^ [24] in kinase buffer (20mM Tris/HCl, 10mM MgCl_2_, 25mM NaCl) containing 100µM ATP and 1µCi of radio-labelled ATP. The kinase reactions were performed during 15min at 25°C, stopped by addition of one volume of 2× Laemmli buffer and boiled 2min. The samples were analysed by SDS-PAGE. The gels were stained by Coomassie dye, dried, and the phosphorylated proteins were revealed using a Phosphoimager (Molecular Dynamics).

### Immunofluorescence

HeLa and U2OS cells grown on coverslips were fixed in methanol at -20°C and processed for immunofluorescence following standard protocols (Montembault et al., 2007). Cell preparations were visualised with a Leica DMRXA2 microscope (objective 63×, N.A. 1,32). Measurements of fluorescence intensities and image acquisition were performed using a CooSnap ES camera with Metamorph software (Roper Scientific), and pictures were prepared as single sections or maximum intensity projections. Alternatively, slides were viewed with a Leica DMIRE2 (SP2) inverted confocal microscope (objective 63×, N.A. 1,4) using the LCS 3D software (Leica). Images were processed with Adobe Photoshop (v7.0).

### Purification of recombinant proteins

MPB and MBP-CDK11^P58^ protein immobilisation on amylose column (Biolabs) was described before [35]. Plk4-V5-(His)6 expression was induced in *E. coli* for 4 hours at 25°C and the recombinant protein was purified following standard procedures [36] using Ni-NTA agarose beads (Quiagen). The Plk4-V5-(His)6 protein was stored at −20°C in 50% glycerol before using for the *in vitro* MBP-CDK11^P58^ binding assay.

### In vitro binding assay

10µg of Maltose Binding Protein (MBP) or MPB-CDK11^p58^ were immobilised on amylose column (stored at 4°C in column buffer: 20mM Tris-HCL pH 7,4; 200mM NaCl; 1mM EDTA and 1mM Sodium Azide) and incubated with 5µg of purified Plk4-V5-(His)6 protein kinase in binding buffer (PBS; 0,1% triton X-100 and 0,1% NP40) for 1h at 4°C. After 3 washes for 5min at 4°C with 1 ml of binding buffer, the beads content was then eluted by Laemmli buffer and the samples were analysed by anti-V5 Western blot.

## Supporting Information

Figure S1 Localisation of Myc-tagged CDK11p58 in HeLa cells. A) HeLa stable cell line was induced to express Myc-CDK11^p58^ for approximately 2 hours, fixed and stained for a-tubulin (red in the left panels and monochrome in the middle panels) and Myc (green in the left panels and monochrome in the right panels). DNA is blue. Each phase of the cell cycle is indicated at the top of each panel. During interphase (top panels), CDK11 remained cytoplasmic and started to accumulate on the astral microtubules during prophase. The tagged protein remained associated with spindle microtubules throughout the duration of cell division. During cytokinesis a pool of the protein was found at the midbody. The mitotic phases are displayed on the merge panels. Scale bar is 10 µm. B) Interphase HeLa cell expressing Myc-CDK11^p58^. The lower panels show a 10 times magnification of the centrosomal region. C) Mitotic HeLa cell expressing Myc-CDK11^p58^. The lower panels show a 5 times enlargement of one of the spindle pole region. In panels B and C, Myc-CDK11^p58^ is green and monochrome in the right panel; pericentrin is red and monochrome in the middle panel; and DNA is blue. Scale bars are 10 mm. See the complete absence of Myc signal at the centrosome in interphase cell and the moderate signal at the spindle pole region in mitotic cells.
(4.43 MB TIF)

Figure S2 CDK11^p58^ depletion interferes with centrosome overduplication in S phase-arrested U2OS cells. A) U2OS cells were transfected with *control*, *Plk4*, or *CDK11* siRNAs. 24 hours post-transfection (duration of one cell cycle), the cells were treated with aphidicolin for 48 hours to be arrested in S phase. Cells were then fixed and the centrosomes were counted. B) The top panel shows an S phase-arrested cell with more than two centrosomes (white arrowhead). The bottom panel shows a cell with two centrosomes (white arrowhead). DNA is blue and γ tubulin, as a centrosomal marker, is red and also displayed in monochrome on the right panels. Scale bar is 10µm. C) Graph (±SD) showing the percentage of cells with more than two centrosomes, the siRNA treatment is indicated at the bottom. Note the 3-fold decrease of centriole overduplication following *Plk4* and *CDK11* RNAi.
(0.70 MB TIF)

Figure S3 The centrosomal recruitment of Cep192 in mitosis is compromised in CDK11-depleted cells. A) *Control* (top) or *CDK11* siRNA-transfected (middle and lower panels) mitotic cells were fixed and stained for DNA (blue), a tubulin (red) and Cep192 (green and right panels in monochrome). Scale bar is 10 µm. B) Graph showing Cep192 signal intensity (±SD) at the mitotic centrosomes of control (green) and CDK11-depleted cells (red).
(1.05 MB TIF)

Figure S4 CDK11^p58^ is not a Plk4 substrate *in vitro*. β casein, Maltose binding protein (MBP) and MBP-CDK11^p58^ were incubated without (lanes 2, 3 and 4 respectively) or with active GST-PLK4 (lanes 5, 6 and 7 respectively) in the presence of radiolabelled ATP. β casein is phosphorylated but not MBP or MBP-CDK11^p58^. The dark arrowheads indicate the positions of the recombinant proteins. Auto-phosphorylation of GST-Plk4 (protein not visible by Coomassie staining) is detected on the autoradiography (lanes 1, 5, 6 and 7) and is indicated by an arrow.
(0.66 MB TIF)

Figure S5 Possible model of centriole duplication. Centriole disengagement (licensing) is controlled by Plk1 and separase to allow subsequent centriole duplication in interphase. Plk1 protein kinase controls centrosome maturation during early mitosis (by recruitment of PCM proteins including SPD-2/Cep192) and centriole disengagement. Separase participates to this process in late mitosis. Plk1 recruitment is under the control of CDK11^p58^ protein kinase, which is only expressed during mitosis. Plk4 plays a key role for centriole duplication during interphase and enhances this process when overexpressed. Plk4 protein accumulation to the mitotic centrosome, is controlled by CDK11^p58^. Both proteins interacts directly with each other indicating a possible involvement of the Plk4 protein kinase during mitosis in the licensing process.
(0.66 MB TIF)

Table S1 Analysis of mitotic figures following CDK11, Plk4, and double RNAi in HeLa cells. The mitotic cells were fixed and analysed for their mitotic spindle shape and their centriole numbers (a scheme of each mitotic figure is displayed at the top of each column). The percentage of each mitotic figure is indicated (±SD).
(0.05 MB DOC)
