# Supplementary figures and images for: CDK11p58 Is Required for Centriole Duplication and Plk4 Recruitment to Mitotic Centrosomes
Source: PLoS One. 2011 Jan 31;6(1):e14600. doi: 10.1371/journal.pone.0014600 (PMC3031510; doi:10.1371/journal.pone.0014600)

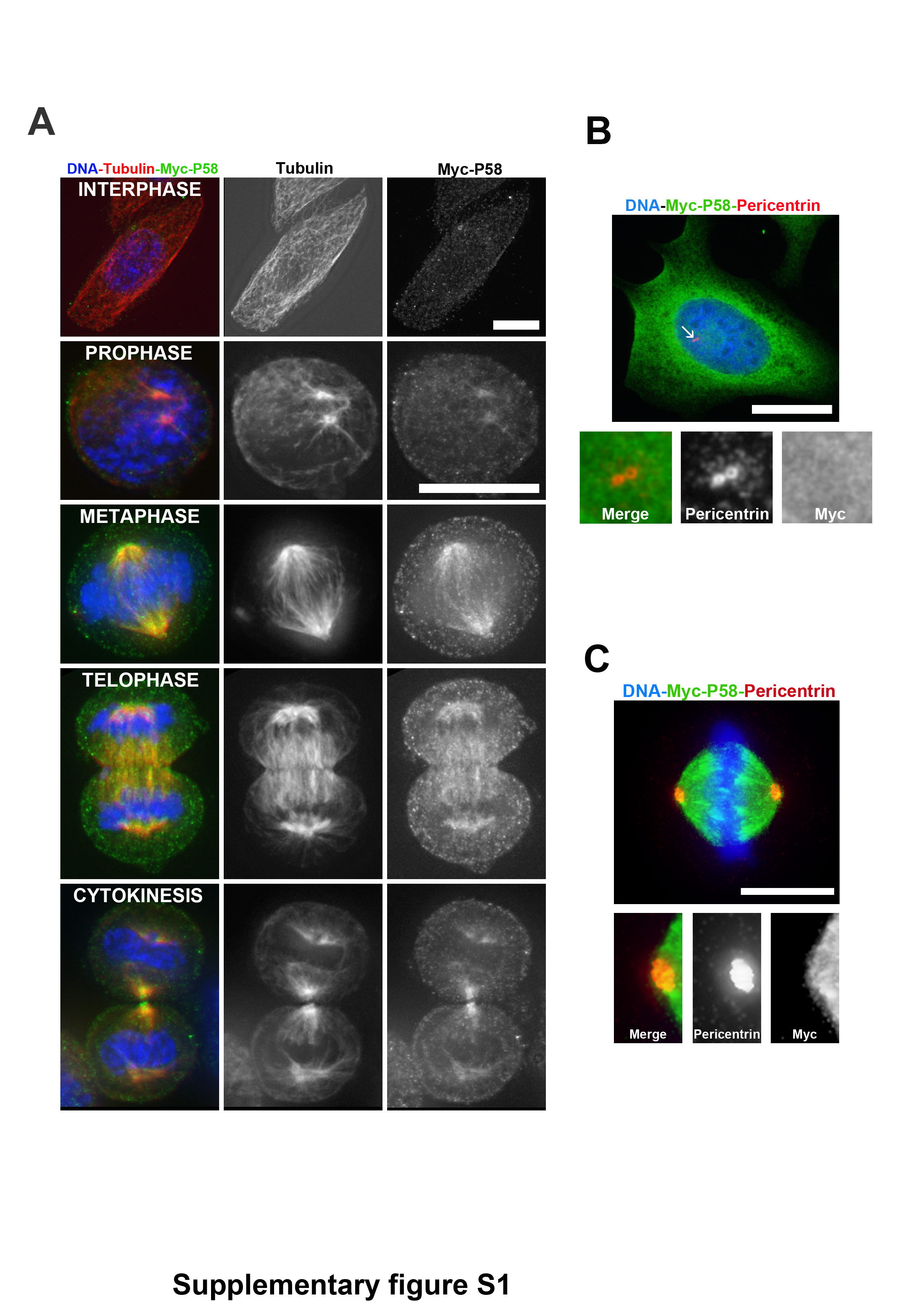

Supplement: Figure S1 — Localisation of Myc-tagged CDK11p58 in HeLa cells. A) HeLa stable cell line was induced to express Myc-CDK11p58 for approximately 2 hours, fixed and stained for a-tubulin (red in the left panels and monochrome in the middle panels) and Myc (green in the left panels and monochrome in the right panels). DNA is blue. Each phase of the cell cycle is indicated at the top of each panel. During interphase (top panels), CDK11 remained cytoplasmic and started to accumulate on the astral microtubules during prophase. The tagged protein remained associated with spindle microtubules throughout the duration of cell division. During cytokinesis a pool of the protein was found at the midbody. The mitotic phases are displayed on the merge panels. Scale bar is 10 µm. B) Interphase HeLa cell expressing Myc-CDK11p58. The lower panels show a 10 times magnification of the centrosomal region. C) Mitotic HeLa cell expressing Myc-CDK11p58. The lower panels show a 5 times enlargement of one of the spindle pole region. In panels B and C, Myc-CDK11p58 is green and monochrome in the right panel; pericentrin is red and monochrome in the middle panel; and DNA is blue. Scale bars are 10 mm. See the complete absence of Myc signal at the centrosome in interphase cell and the moderate signal at the spindle pole region in mitotic cells. (4.43 MB TIF) [file pone.0014600.s001.tif]

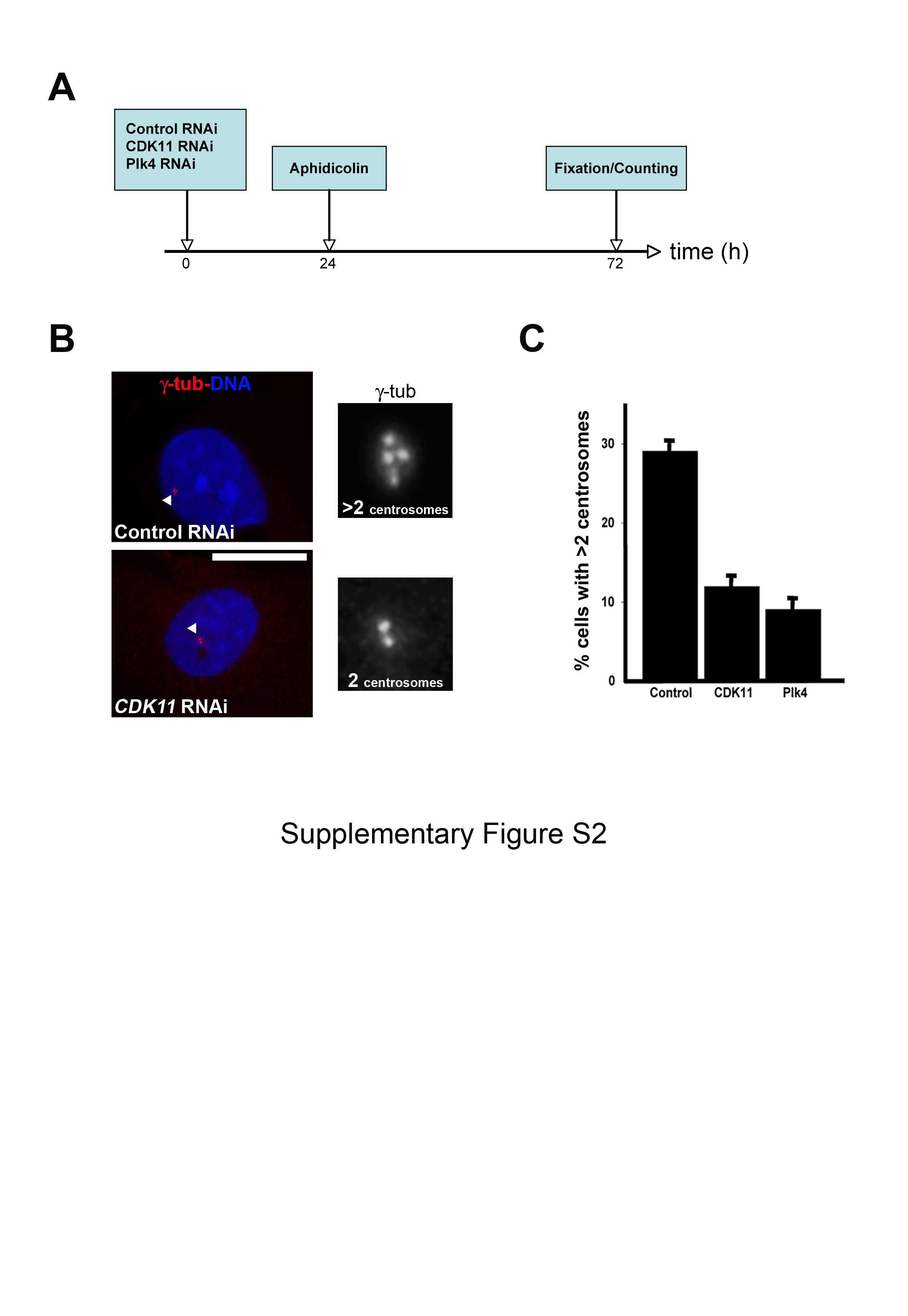

Supplement: Figure S2 — CDK11p58 depletion interferes with centrosome overduplication in S phase-arrested U2OS cells. A) U2OS cells were transfected with control, Plk4, or CDK11 siRNAs. 24 hours post-transfection (duration of one cell cycle), the cells were treated with aphidicolin for 48 hours to be arrested in S phase. Cells were then fixed and the centrosomes were counted. B) The top panel shows an S phase-arrested cell with more than two centrosomes (white arrowhead). The bottom panel shows a cell with two centrosomes (white arrowhead). DNA is blue and γ tubulin, as a centrosomal marker, is red and also displayed in monochrome on the right panels. Scale bar is 10µm. C) Graph (±SD) showing the percentage of cells with more than two centrosomes, the siRNA treatment is indicated at the bottom. Note the 3-fold decrease of centriole overduplication following Plk4 and CDK11 RNAi. (0.70 MB TIF) [file pone.0014600.s002.tif]

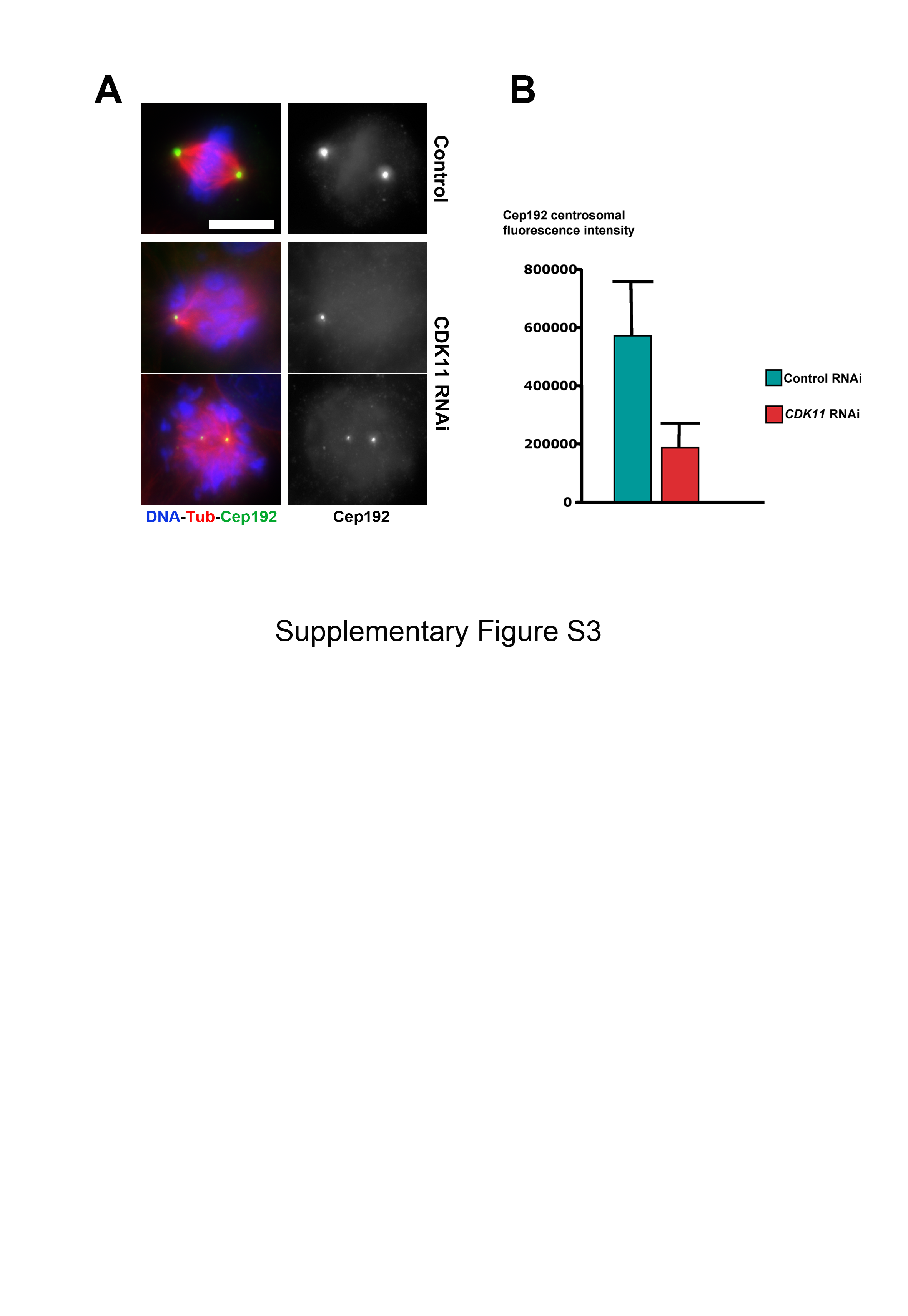

Supplement: Figure S3 — The centrosomal recruitment of Cep192 in mitosis is compromised in CDK11-depleted cells. A) Control (top) or CDK11 siRNA-transfected (middle and lower panels) mitotic cells were fixed and stained for DNA (blue), a tubulin (red) and Cep192 (green and right panels in monochrome). Scale bar is 10 µm. B) Graph showing Cep192 signal intensity (±SD) at the mitotic centrosomes of control (green) and CDK11-depleted cells (red). (1.05 MB TIF) [file pone.0014600.s003.tif]

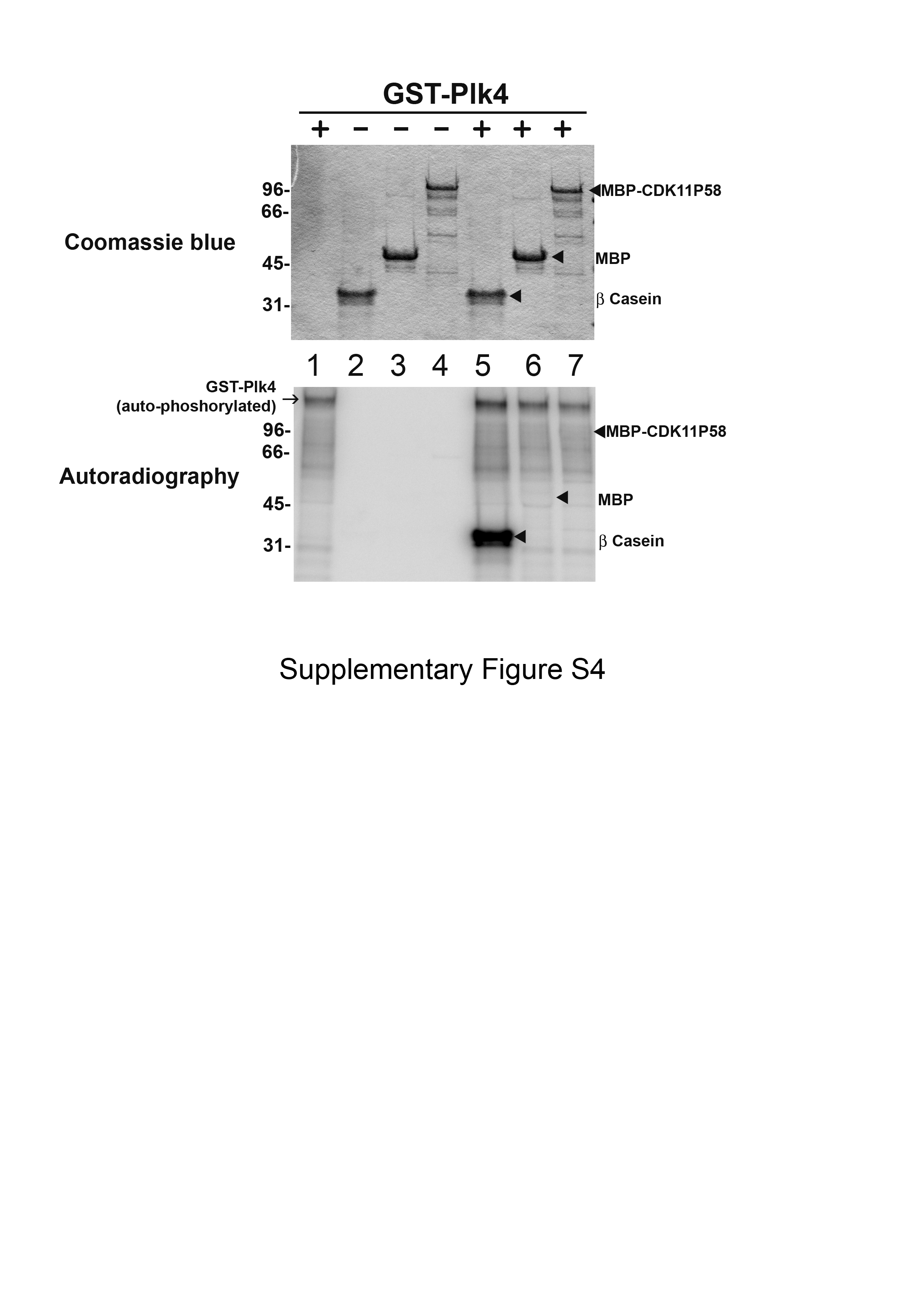

Supplement: Figure S4 — CDK11p58 is not a Plk4 substrate in vitro. β casein, Maltose binding protein (MBP) and MBP-CDK11p58 were incubated without (lanes 2, 3 and 4 respectively) or with active GST-PLK4 (lanes 5, 6 and 7 respectively) in the presence of radiolabelled ATP. β casein is phosphorylated but not MBP or MBP-CDK11p58. The dark arrowheads indicate the positions of the recombinant proteins. Auto-phosphorylation of GST-Plk4 (protein not visible by Coomassie staining) is detected on the autoradiography (lanes 1, 5, 6 and 7) and is indicated by an arrow. (0.66 MB TIF) [file pone.0014600.s004.tif]

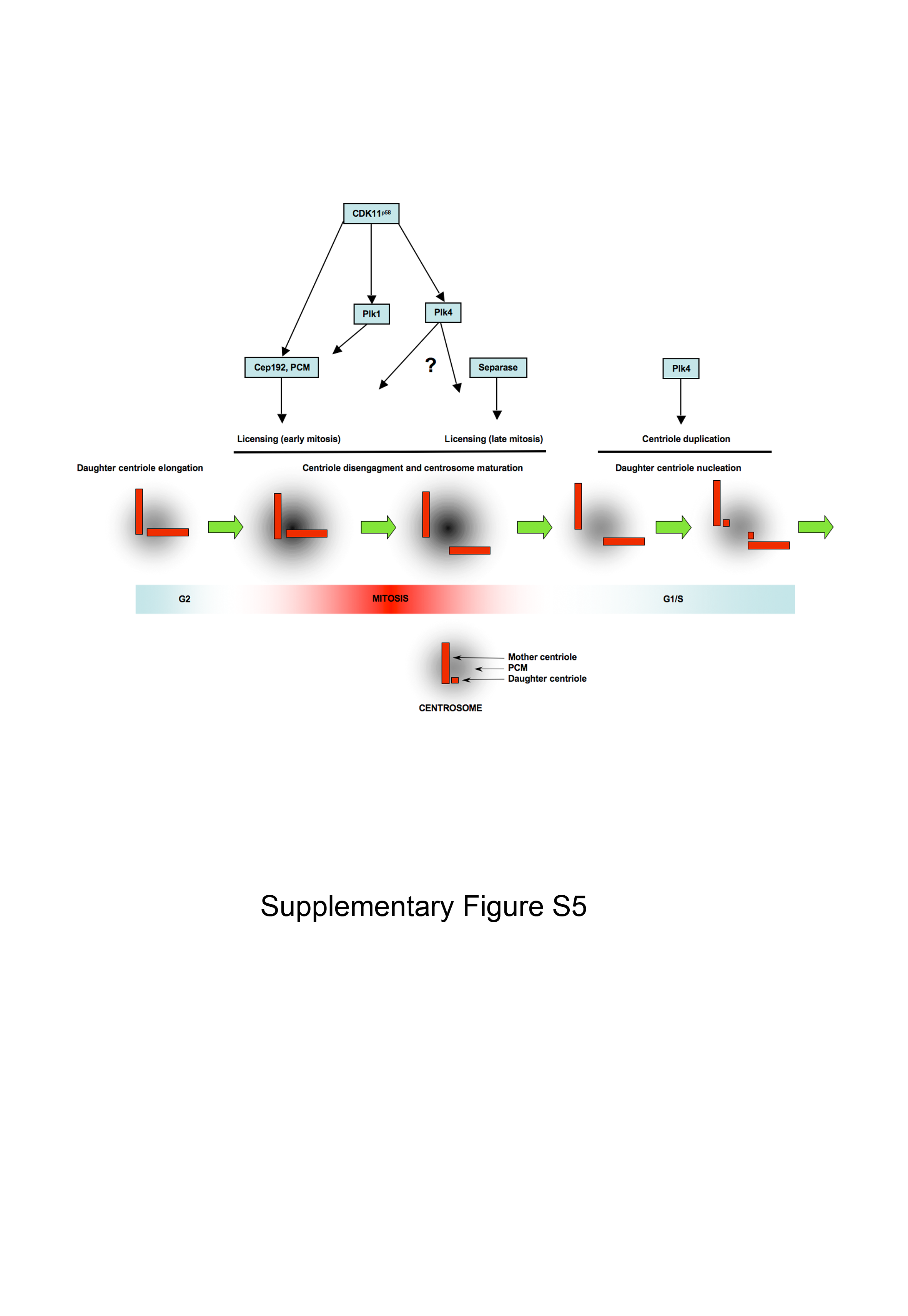

Supplement: Figure S5 — Possible model of centriole duplication. Centriole disengagement (licensing) is controlled by Plk1 and separase to allow subsequent centriole duplication in interphase. Plk1 protein kinase controls centrosome maturation during early mitosis (by recruitment of PCM proteins including SPD-2/Cep192) and centriole disengagement. Separase participates to this process in late mitosis. Plk1 recruitment is under the control of CDK11p58 protein kinase, which is only expressed during mitosis. Plk4 plays a key role for centriole duplication during interphase and enhances this process when overexpressed. Plk4 protein accumulation to the mitotic centrosome, is controlled by CDK11p58. Both proteins interacts directly with each other indicating a possible involvement of the Plk4 protein kinase during mitosis in the licensing process. (0.66 MB TIF) [file pone.0014600.s005.tif]
