# Supplementary material for: CDK11p58 Is Required for Centriole Duplication and Plk4 Recruitment to Mitotic Centrosomes
Source: PLoS One. 2011 Jan 31;6(1):e14600. doi: 10.1371/journal.pone.0014600 (PMC3031510; doi:10.1371/journal.pone.0014600)
Supplement: Table S1 — Analysis of mitotic figures following CDK11, Plk4, and double RNAi in HeLa cells. The mitotic cells were fixed and analysed for their mitotic spindle shape and their centriole numbers (a scheme of each mitotic figure is displayed at the top of each column). The percentage of each mitotic figure is indicated (±SD). (0.05 MB DOC) [file pone.0014600.s006.doc]

**Supplementary Table 1. Analysis of mitotic figures following CDK11, Plk4, and double RNAi in HeLa cells**

| RNAi treatment |  |  |  |  |  |  |  |  |  |  | >4 centriole  during prophase |
| --- | --- | --- | --- | --- | --- | --- | --- | --- | --- | --- | --- |
| Control | 90,6±1,9 | 0 | 0 | 0 | 0,5±1,0 | 2,2±1,8 | 0 | 0 | 0 | 0 | 6,7±1,9 |
| CDK11 | 10,8±4,1 | 19,1±2,1 | 1,8±1,1 | 0 | 6,0±2,1 | 0,2±0,5 | 4,4±4,8 | 13,9±5,1 | 36,1±4,3 | 4,2±1,5 | 3±1,3 |
| Plk4 | 14,4±9,0 | 9,0±4,7 | 8,6±5,3 | 6,3±3,0 | 11,6±6,9 | 3,3±2,0 | 5,7±3,0 | 17,8±12,8 | 17,3±8,1 | 3,5±3,4 | 3,9±1,9 |
| Plk4+CDK11 | 1,5±1,3 | 10,2±1,6 | 2,0±0,7 | 3,5±0,9 | 2,4±1,2 | 0 | 3,1±0,8 | 67,8±2,6 | 6,9±2,1 | 1,1±1 | 1,5±0,6 |
